# Cysteine depletion sensitizes prostate cancer cells to agents that enhance DNA damage and to immune checkpoint inhibition

**DOI:** 10.1186/s13046-023-02677-2

**Published:** 2023-05-11

**Authors:** Achinto Saha, Shengyuan Zhao, Austin Kindall, Carly Wilder, Chelsea A. Friedman, Rachel Clark, George Georgiou, Everett Stone, Dawit Kidane, John DiGiovanni

**Affiliations:** 1 Division of Pharmacology and Toxicology and Dell Pediatric Research Institute, The University of Texas at Austin, 1400 Barbara Jordan Blvd, Austin, TX 78723 USA; 2 Department of Chemical Engineering, The University of Texas at Austin, Austin, TX 78712 USA; 3 Department of Molecular Biosciences, The University of Texas at Austin, Austin, TX 78712 USA; 4 Institute for Cellular and Molecular Biology, The University of Texas at Austin, Austin, TX 78712 USA; 5 Center for Molecular Carcinogenesis and Toxicology, The University of Texas at Austin, Austin, TX 78712 USA; 6 LiveSTRONG Cancer Institutes, Dell Medical School, The University of Texas at Austin, Austin, TX 78712 USA; 7 Department of Phyisiology & Biophysics, College of Medicine, Howard University, 520 W Street, NW, Washington, DC 20059 USA

**Keywords:** Prostate cancer, Reactive Oxygen Species, DNA damage, Combination therapy, Immune checkpoint inhibitor

## Abstract

**Background:**

Prostate Cancer (PCa) represents one of the most commonly diagnosed neoplasms in men and is associated with significant morbidity and mortality. Therapy resistance and significant side effects of current treatment strategies indicate the need for more effective agents to treat both androgen-dependent and androgen-independent PCa. In earlier studies, we demonstrated that depletion of L-cysteine/cystine with an engineered human enzyme, Cyst(e)inase, increased intracellular ROS levels and inhibited PCa growth in vitro and in vivo. The current study was conducted to further explore the mechanisms and potential combinatorial approaches with Cyst(e)inase for treatment of PCa.

**Methods:**

DNA single strand breaks and clustered oxidative DNA damage were evaluated by alkaline comet assay and pulsed field gel electrophoresis, respectively. Neutral comet assay and immunofluorescence staining was used to measure DNA double strand breaks. Cell survival and reactive oxygen species level were measured by crystal violet assay and DCFDA staining, respectively. Western blot was used to determine protein expression. FACS analyses were preformed for immune cell phenotyping. Allograft and xenograft tumor models were used for assessing effects on tumor growth.

**Results:**

PCa cells treated with Cyst(e)inase lead to DNA single and double strand breaks resulted from clustered oxidative DNA damage (SSBs and DSBs). Cyst(e)inase in combination with Auranofin, a thioredoxin reductase inhibitor, further increased intracellular ROS and DNA DSBs and synergistically inhibited PCa cell growth in vitro* and *in vivo. A combination of Cyst(e)inase with a PARP inhibitor (Olaparib) also increased DNA DSBs and synergistically inhibited PCa cell growth in vitro* and *in vivo without additional ROS induction. Knockdown of BRCA2 in PCa cells increased DSBs and enhanced sensitivity to Cyst(e)inase. Finally, Cyst(e)inase treatment altered tumor immune infiltrates and PD-L1 expression and sensitized PCa cells to anti-PD-L1 treatment.

**Conclusions:**

The current results demonstrate the importance of oxidative DNA damage either alone or in combination for Cyst(e)inase-induced anticancer activity. Furthermore, cysteine/cystine depletion alters the tumor immune landscape favoring enhanced immune checkpoint inhibition targeting PD-L1. Thus, combinatorial approaches with Cyst(e)inase could lead to novel therapeutic strategies for PCa.

**Supplementary Information:**

The online version contains supplementary material available at 10.1186/s13046-023-02677-2.

## Background

PCa is the most common non-skin cancer and the second leading cause of cancer-related death in men in the United States, with a projected 268,490 new cases and an estimated 34,500 deaths in 2022 [1]. The reliance of PCa cells on androgen for their growth provides successful treatment option for patients with androgen deprivation therapy. However, the disease can eventually progress to an androgen-independent state known as castrate resistant prostate cancer (CRPC) and becomes unresponsive to chemotherapy, radiotherapy or hormonal therapy [2–4]. Second generation anti-androgens (e.g., abiraterone or enzalutamide), have provided modest survival benefits of ~ 4–5 months [5, 6], however, these agents eventually fail due to the development of resistance. Moreover, as chemotherapy related toxicities seriously compromise the quality of life of patients, the identification of newer agents with better efficacy and less toxicity are urgently needed that effectively treat both androgen-dependent and androgen-independent PCa.

Cancer cells experience consistently higher oxidative stress generated by reactive oxygen species (ROS) (reviewed in [7]). Mechanisms responsible for increased oxidative stress in cancer cells include genetic alterations (activation of oncogenes such as *c-Myc, Ras* or *Bcl-abl*, loss of p53), active energy metabolism associated with uncontrolled cell proliferation, malfunction of mitochondrial respiration associated with mtDNA mutations [8–10] and an inflammatory microenvironment that causes a significant increase in ROS levels relative to non-malignant tissues [9, 11]. High levels of ROS can lead to oxidative DNA damage that can be detrimental to cancer cell survival [9]. Thus, cancer cells must maintain optimal levels of ROS for growth and survival. Glutathione (GSH) is the major antioxidant system responsible for maintaining cellular redox potential essential for normal cellular function and viability. Cancer cells have high demand for antioxidants such as GSH and they achieve this oxidative balance through the upregulation of antioxidant defense mechanisms [12]. This provides a unique opportunity to selectively kill cancer cells by targeting these antioxidant defense mechanisms. In this regard, we recently reported that depletion of L-cysteine (L-Cys) and cystine (CSSC) with a novel engineered human enzyme, Cyst(e)inase, leads to near complete depletion of intracellular GSH, increased intracellular ROS levels and selective inhibition of cancer cell survival in a wide range of cancer types, including PCa [13, 14].

Here, we report that depletion of L-Cys and CSSC by Cyst(e)inase leads to DNA damage, including clustered oxidative DNA lesions and DNA single strand and double strand breaks (SSBs and DSBs, respectively) in PCa cells. In addition, Cyst(e)inase in combination with Auranofin [a thioredoxin reductase (TXNR) inhibitor] or Olaparib [a poly (ADP-ribose) polymerase (PARP) inhibitor] provided synergistic increases in DNA damage, inhibition of cancer cell survival and inhibition of tumor growth in vivo. Furthermore, BRCA2 deficiency also sensitized PCa cells to L-Cys depletion. Finally, Cyst(e)inase was also found to sensitize PCa cells to immune checkpoint inhibition with anti-programmed cell death ligand -1 (anti-PD-L1) antibody. Taken together, the current data suggest that enhancement of Cyst(e)inase-induced DNA damage and synergistic PCa tumor cell killing can be achieved through combinatorial approaches or targeting tumors with certain mutations. Furthermore, L-Cys and CSSC depletion also favorably alters the immune landscape allowing synergistic tumor inhibition with immune checkpoint inhibitors anti-PD-L1. These results suggest potential new therapeutic strategies for the treatment of PCa.

## Methods

### Cell culture

Human PCa cell lines 22Rv1 (representative cell line of castrate resistant prostate cancer, derived from the xenografted tumor in castrated mouse inoculated with androgen dependent CWR22 cells) and PC3 (derived from the bone metastasis of prostate adenocarcinoma) were purchased from the American Type Culture Collection (ATCC; Manassas, VA). Murine PCa cell line HMVP2, a relevent model of human PCa, were developed in house from a ventral prostate tumor of 1 year old HiMyc mice [15]. This PCa cell line possesses stem cell like properties and express various stem cell markers such as CD49f, Sca-1, CK14 and CD29 [15]. Cells were grown in RPMI-1640 containing 10% FBS. All cell lines were supplemented with 1% penicillin/streptomycin, tested negative for mycoplasma by PCR analysis (Applied Biological Materials Inc.) and cultured at 37 °C in 5% CO2 incubator.

### Western blotting

Western blotting was performed as described previously [16]. Briefly, cells or tumors were lysed with RIPA buffer supplemented with protease inhibitor (#25,765,800, Sigma) and phosphatase inhibitor (#P5726, Sigma) for 30 min. 30 μg of lysates were separated by SDS-PAGE and transferred to nitrocellulose membrane, blocked with 5% BSA for 1 h at room temperature, then incubated with primary antibody overnight at 4 °C. Primary antibodies include: BRCA2 (#ab27976, Abcam); γ-H2AX ^S139^ (#05–636, Millipore, 1:1000); H2AX (#2595, Cell Signaling, 1:1000); α-Tubulin (#2144, Cell Signaling, 1:1000); Actin (#A5316, Sigma 1:30,000); p-Chk1^S345^(#2348, Cell Signaling, 1:1000); Chk1 (#2360, Cell Signaling, 1:1000); p-p53^Ser15^(#82,530, Cell Signaling, 1:1000); p53(#2524, Cell Signaling, 1:1000); p-ATM^Ser1981^(#4526, Cell Signaling, 1:1000); ATM (#2873, Cell Signaling, 1:1000); PD-L1 (#ab213480, Abcam, 1:1000). The next day, membranes were washed with PBST (0.1% Tween-20) three times, incubated with secondary antibody for 2 h at room temperature before developing with ECL substrate (#32,106, Thermo Fisher).

### Immunofluorescence

Cells were grown on chamber slides overnight prior to drug treatment, fixed in 3.7% formaldehyde in PBS for 10 min at room temperature, permeabilized in 0.5% Triton X-100 for 15 min and blocked in 3% BSA for 1 h at room temperature. Cells were incubated with primary antibody overnight at 4 °C and secondary antibody for 1 h at room temperature. Slides were mounted with DAPI (#H-1200, Vector Laboratories) and image were acquired using Zess confocal microscope.

### Small interfering RNA (siRNA) for knockdown of BRCA2

ON-TARGETplus Human and mouse BRCA2 siRNA SMARTpool (Cat. L-003462–00-0005, L-042993–00-0005) were purchased from Dharmacon Research Inc. All siRNA was suspended in RNase-free water at a concentration of 5 μM. A final siRNA concentration of 25 nM was used for subsequent experiments and knockdown of BRCA2 was performed according to manufacturer instructions.

### Cell survival assay

Cells were seeded at a density of 1000–10,000 cells per well for 3 days’ drug treatment in 96-well plates. 10 μl of MTT solution (12 mM) was added to each well, and the plates were incubated at 37 °C for 2 h in the dark. After incubation, medium of each well was replaced with 100 μl DMSO. Plates were then incubated at 37 °C for another 10 min, and the absorbance was measured at 570 nm. Alternatively, cell survival was further confirmed by crystal violet assay [14].

### DNA comet assay

Comet assay was performed using Comet Assay Kit (#4250–050-K, Trevigen). 1,000 cells were mixed with LMA agarose at 37 °C, and the mixture was immediately added to the slides. After drying the slides at 4 °C for 10 min, cells were lysed with lysis buffer (#4250–050-01, Trevigen) at 4 °C overnight. Alkali comet assay (pH > 13) and Neutral comet assay (pH = 9) were performed according to the provided protocol of the kit. Slides were then stained with SYBR Gold (#S11494, Invitrogen) for 30 min at room temperature, and visualized under 20X objective lens.

### Pulse field gel electrophoresis

The fraction of fragmented DNA assay allows detection of Cyst(e)inase‐induced clustered oxidative DNA damage and formamidopyrimidine‐DNA glycosylase (Fpg)‐revealed DSBs present in the whole genome of the cells and was performed as described previously [17]. Briefly, cells were trypsinized and mixed with CleanCut Agarose from CHEF Mammalian Genomic DNA Plug Kit (#1,703,591, Bio-rad). Each plug contained 100,000 cells. Plug was lysed with proteinase K at 4 °C overnight, washed four times with 1X washing buffer. For FPG digestion, 100 mM PMSF was added in the second round of wash, and plug was later incubated with 20 units FPG (#M0240S, New England Biolabs) at 37 °C for 3 h. Electrophoresis was performed using CHEF-DR II System (#1,703,725, Bio-Rad) at 14 °C with 60–120 s for switch time, 24 h for run time, 6 V/cm for voltage gradient. Saccharomyces cerevisiae chromosome (#170–3605, Bio-Rad) was used as DNA ladder. Gels were then stained with ethidium bromide for 30 min before visualization. Data analysis in fold change were carried out by taking the difference in the absolute number of measured intensity of DNA fragment that migrate in the agarose gel during PFGE in Cyst(e)inase/FPG treated and divided to that in the control (Cyst(e)inase/ FPG untreated group). Note that the value for the background staining in each lane has been subtracted in the fold change calculations. Images were taken using ChemiDoc™ System (Bio-Rad). All intensity measurement were carried out using Image J software.

### Measurement of reactive oxygen species

The intracellular reactive oxygen species (ROS) was measured using 2′,7′-dichlorofluorescin diacetate (DCFDA) fluorescence. Cells in a 96 well plate were treated with indicated concentrations of Cyst(e)inase, Olaparib/Auranofin or their combinations for 24 h, stained with DCFDA (20 µM; Sigma) at 37 °C for 2 h and then fluorescence intensity was measured at the respective excitation and emission wavelengths of 485 nm and 535 nm using a fluorescent plate reader (Tecan Group Ltd.).

### Animal experiments

All protocols were approved by the Institutional Animal Care and Use Committee of the University of Texas at Austin. All mice were allowed to acclimate for at least 1 week prior to use in experiments. For all tumor studies, tumor size was measured 2–3 times weekly using a digital caliper. Tumor volume was calculated by the formula: 0.5236 D1(D2)^2^, where D1 and D2 are the long and short diameter, respectively. Mice were given a semi-purified diet (AIN76A, 10 kcal%, Research Diets) and water ad libitum. Food consumption and body weight of the mice were measured weekly. Experiments were terminated when tumor sizes in the control group reached their maximum limit as specified by the protocol.

#### 22Rv1 xenograft tumors

22Rv1 cells (2 × 10^6^) were mixed with matrigel (1:1) (100 uL cell suspension in serum free media + 100 uL matrigel) and injected subcutaneously into both flanks of male 6–7 weeks old athymic nude mice (outbred homozygous Foxn1nu/ Foxn1nu; J:NU 007,850, Jackson Laboratory). After the tumors were palpable, mice were divided into groups such that the average tumor volumes in all the groups were approximately equal. Treatment was initiated with intraperitoneal (ip) injection of one of the following: a) inactivated Cyst(e)inase (control), b) Cyst(e)inase alone (25 mg/kg, 2x/week), c) Olaparib alone (50 mg/kg, 5/week), d) Cyst(e)inase (25 mg/kg, 2x/week) plus Olaparib (50 mg/kg 5x/ week), e) Auranofin alone (1 mg/kg, 3/week), f) Cyst(e)inase (25 mg/kg, 2x/week) plus Auranofin (1 mg/kg 3x/ week).

#### HMVP2 allograft tumors

Six-7 weeks old male FBV/N mice (Charles River) were injected subcutaneously with 5 × 10^6^ HMVP2 cells into both flanks. When tumors were palpable, mice were divided into 4 groups with approximately equal average tumor volumes and treatments as follows: 1) Control (vehicle); 2) Cyst(e)inase, 25 mk/kg 2 × per week; 3) Anti-PDL1 5 mg/kg 2 × per week 2 weeks; or 4) Cyst(e)inase + Anti-PDL1.

### Statistical analyses

Data are reported as mean ± SEM. Statistical analyses were performed using Student’s t-test, One-way ANOVA or repeated measures two-way ANOVA followed by Dunnett’s or Bonferroni’s multiple comparison test. Significance was set at P ≤ 0.05.

## Results

### PCa cells accumulate DNA SSBs and DSBs with Cyst(e)inase treatment

**Fig. 1:**
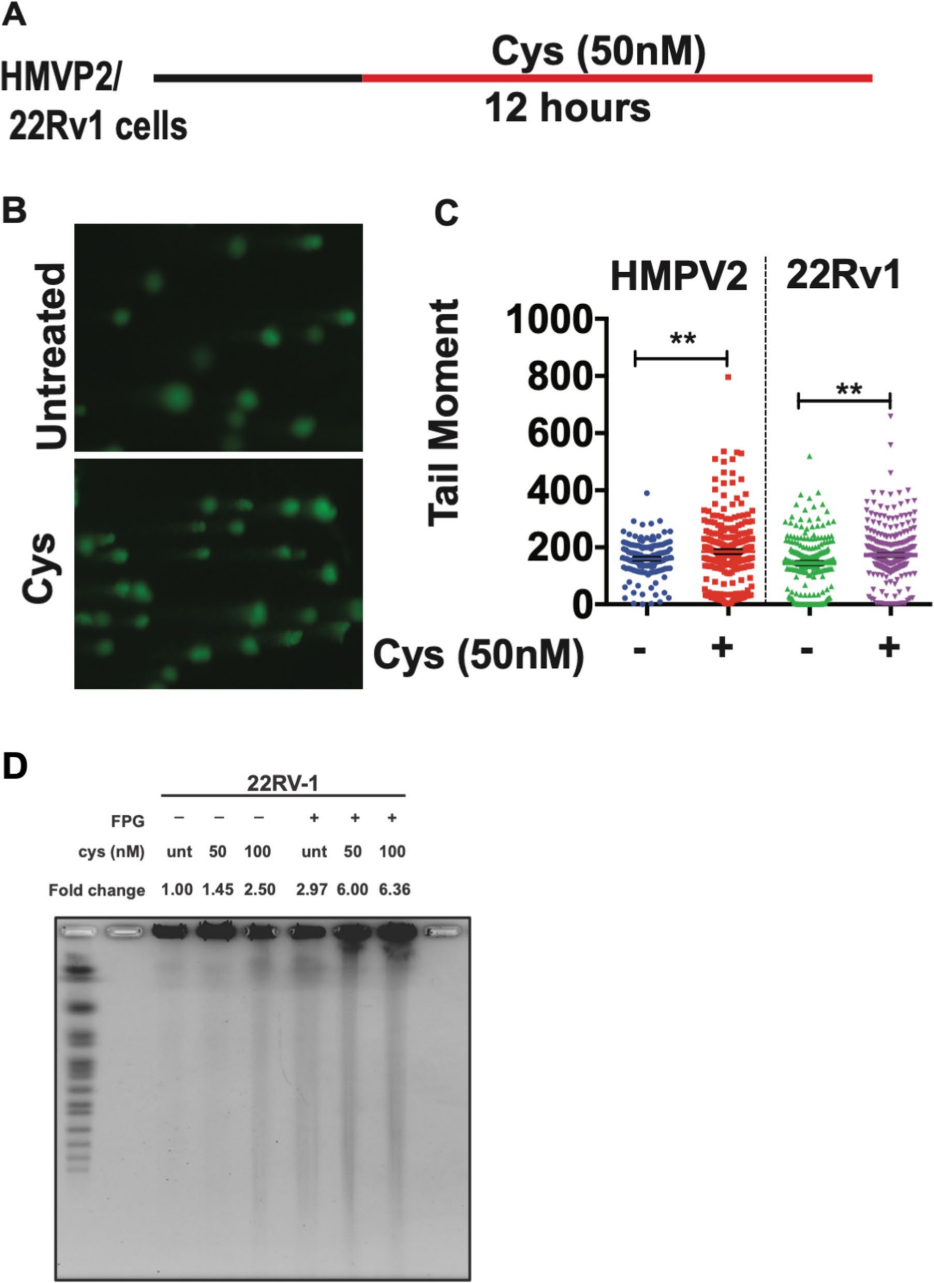
Induction of DNA single strand breaks by Cyst(e)inase. **A** Alkaline comet assay treatment procedure. **B** Representative image of HMVP2 cells treated with vehicle or Cyst(e)inase (50 nM), Scale bar 5 μm; **C** Estimated tail moment in HMVP2 and 22Rv1 PCa cells treated with vehicle or Cyst(e)inase (50 nM) for 12 h; **D** Pulsed Field Gel Electrophoresis after cells (22Rv1) treated with 50 nM Cyst(e)inase. Fold change was calculated by taking the relative intensity of DNA fragment of cells treated with Cyst(e)inase and FPG relative to untreated cells (Image J). **P < 0.01 (Student’s t test) (*n* = 3 independent experiments with at least 150 comets from each group)

We previously reported that treatment of Cyst(e)inase significantly increased ROS levels in several cancer cell lines including PCa cells [13, 14]. Since ROS leads to oxidative DNA damage, we examined whether treatment with Cyst(e)inase induced DNA SSBs and DSBs in PCa cells. As shown in Fig. 1A-C, 12 h after treatment with Cyst(e)inase, both mouse (HMVP2) and human (22Rv1) PCa cells showed significantly higher SSBs compared to untreated cells as measured by the alkaline comet assay. It is already well established that the presence of closely clustered oxidative DNA damage can lead to DNA DSBs. Therefore, we measured the level of clustered oxidative DNA damage in cells treated with Cyst(e)inase for 24 h by pulsed field gel electrophoresis (PFGE). Cells treated with Cyst(e)inase were embedded with low melting soft agar and exposed to FPG glycosylase. The resulting AP sites, if clustered in close proximity, will lead to single-strand nicks on opposing strands, resulting in DSBs. As shown in Fig. 1D, there was a significant increase in DNA fragments (~ 4.4-fold increase) in 22Rv1 cells treated with Cyst(e)inase followed by FPG enzyme treatment indicating increased oxidative clustered DNA lesions formation. The accumulation of clustered oxidative DNA lesions after Cyst(e)inase treatment in PCa cells are indicative of DNA DSBs formation.

**Fig. 2:**
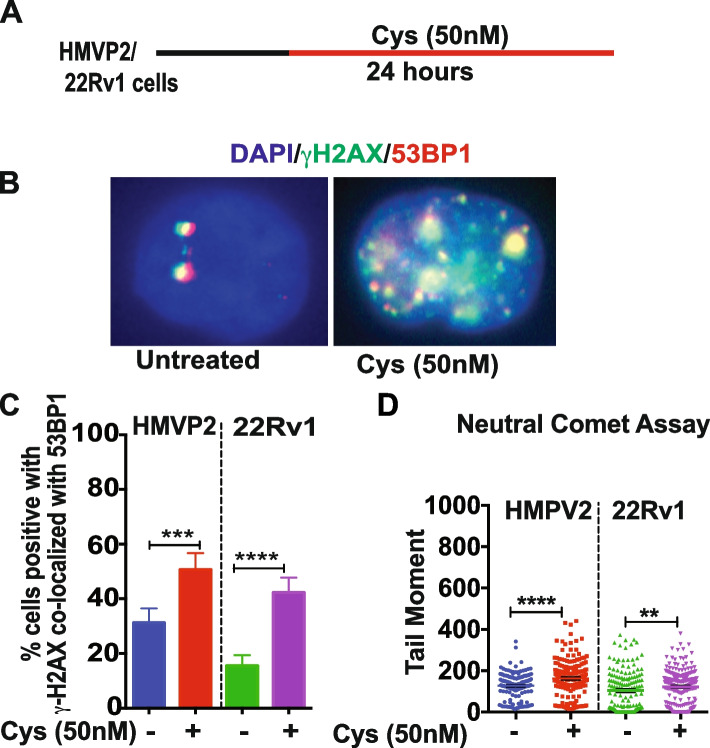
Cyst(e)inase treatment increases DNA double strand breaks. **A** Schematic representation of DNA DSBs experimental procedure; **B** Representative image of γH2AX and 53BP1 co-localization in HMVP2 PCa cells after treatment with vehicle or Cyst(e)inase (50 nM) for 24 h. Scale bar 10 μm; **C** Percentage of cells positive for γH2AX co-localized with 53BP1 24 h after treatment with Cyst(e)inase with more than 100 cells from each cell lines scored; **D** Estimated tail moment using neutral comet assay to measure DNA DSBs after treatment with Cyst(e)inase for 24 h. More than 85 comets were scored. (**P < 0.01, ***P < 0.001, ****P < 0.0001); student’s t-test. *n* = 3 independent experiments

In order to confirm that Cyst(e)inase produced DSBs in PCa cells, we next examined markers of DSBs using immunofluorescence staining to measure the co-localization of γH2AX and 53BP1. As shown in Fig. 2A and B, both mouse and human PCa cells treated with Cyst(e)inase showed increased co-localization of γH2AX/53BP1 compared to untreated cells. The quantitation of γH2AX/53BP1 co-localization showed significantly higher percentage of positive cells with γH2AX co-localized with 53BP1 (Fig. 2C). Neutral comet assay was also performed following treatment of both HMVP2 and 22Rv1 cells with Cyst(e)inase. As shown in Fig. 2D, a significant increase in tail moment was observed confirming increased DNA DSB formation. These data indicate that Cyst(e)inase mediated ROS production as shown previously [13] and clustered oxidative DNA damage (Fig. 1) are associated with the formation of DNA DSBs in PCa cells.

### Combination of L-Cys depletion and TXNR inhibition in PCa cells produces synergistic increases in DNA DSBs

**Fig. 3:**
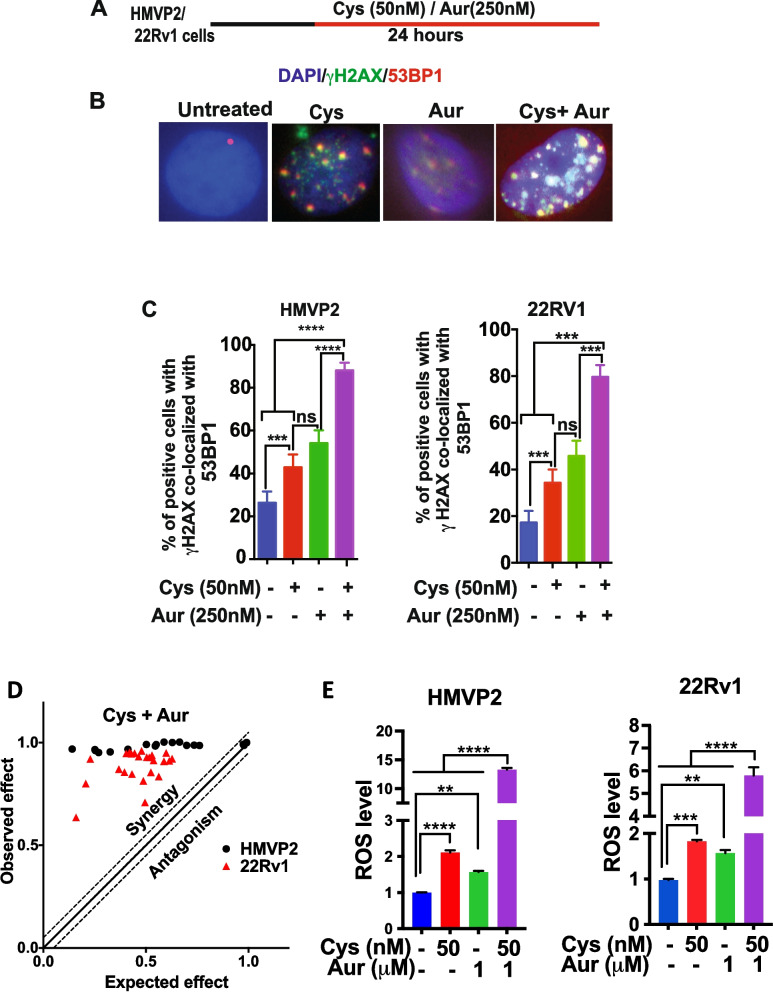
Cyst(e)inase in combination with Auranofin produces synergistic effect to promote DNA DSBs. **A** DNA DSBs treatment procedure. **B** Representative image of γH2AX/53BP1 co-localization in HMVP2 cells after treatment with Cyst(e)inase (50 nM) and Auranofin (250 nM) combination for 24 h, Scale bar 10 μm; **C** Percent of HMVP2 and 22Rv1 cells positive for γH2AX/53BP1 co-localization after cells treated with Cyst(e)inase (50 nM) and Auranofin (250 nM) combination for 24 h with > 100 cells/group included for analysis; **D** Bliss index plot showing synergistic cell survival inhibition in HMVP2 and 22Rv1 cells after treatment with Cyst(e)inase and Auranofin combination; **E** Synergistic induction of ROS in HMVP2 and 22Rv1 cells with combination of Cyst(e)inase and Auranofin. **P < 0.01, ***P < 0.001, ***P < 0.001, One-Way ANOVA followed by Dunnett's multiple comparison test. *n* = 3 independent experiments

We previously reported that a combination of Cyst(e)inase with curcumin that has TXNR inhibitory activity synergistically increased ROS levels and inhibited growth of PCa cells in vitro and in vivo [13]. In more recent studies, we showed that Auranofin, a specific TXNR inhibitor also synergistically increased ROS levels and DNA damage and inhibited growth of PDAC cells in vitro and in vivo [14, 18]. Here, we further investigated whether a combination of Auranofin and Cyst(e)inase could increase oxidative DNA damage and synergistic inhibition of cell growth in PCa cells. As shown in Fig. 3A and B, a combination of Cyst(e)inase and Auranofin treatment significantly increased DNA DSBs in both mouse and human PCa cells. Quantitation of γH2AX and 53BP1 colocalization (Fig. 3C) showed a moderate increase in DSBs with either Cyst(e)inase or Auranofin treatment alone at the concentrations used in both HMVP2 and 22Rv1 cells. However, treatment with the combination of Cyst(e)inase and Auranofin produced significantly higher DSBs in HMVP2 (89%) and 22Rv1 (80%) cells. This was further confirmed by Western blot analysis (Supplemental Fig. 1) showing increased γH2AX levels in both cell lines after treatment with combination of Cyst(e)inase and Auranofin. The combination of Cyst(e)inase and Auranofin also showed a robust increase in phosphorylation of Chk1, p53 and ATM in 22Rv1 cells compared to the individual agents when used alone (Supplemental Fig. 1). We also found that Cyst(e)inase in combination with Auranofin produced strong synergistic inhibition of survival of both mouse and human PCa cell lines (Fig. 3D, Bliss index plot). Finally, as shown in Fig. 3E, the Cyst(e)inase and Auranofin combination produced a dramatic increase in ROS levels (~ 12 and ~ sixfold in HMVP2 and 22Rv1 cells, respectively) compared to cells treated with the individual agents alone.

### Cyst(e)inase in combination with Olaparib produces synergistic DNA damage in PCa cells

**Fig. 4:**
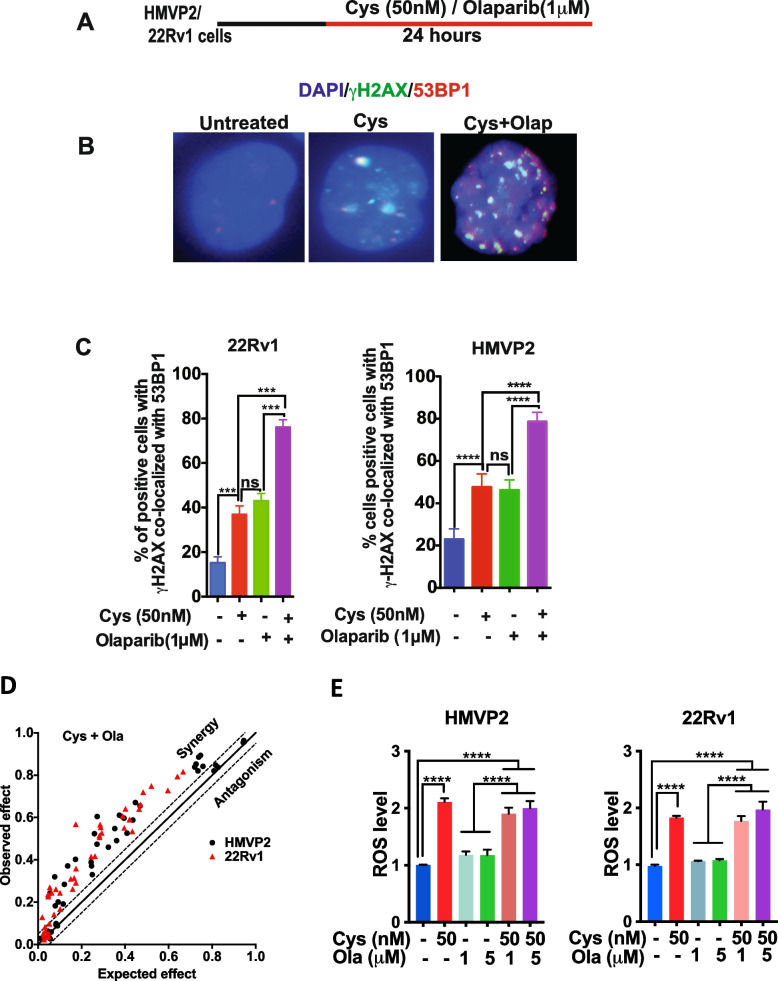
Synergistic induction of DNA DSBs with combination of Cyst(e)inase and Olaparib. **A** DNA DSBs treatment procedure. **B** Representative image of γH2AX/53BP1 co-localization in HMVP2 cells after treatment with Cyst(e)inase (50 nM) and Olaparib (1 μM) combination for 24 h, Scale bar 10 μm; **C** Percent of HMVP2 (left panel) and 22Rv1 (right panel) cells positive for γH2AX/53BP1 co-localization after cells treated with Cyst(e)inase (50 nM) and Olaparib (1 μM) combination for 24 h with > 100 cells/group included for analysis; **D** Bliss index plot showing synergistic cell survival inhibition in HMVP2 and 22Rv1 cells after treatment with Cyst(e)inase and Olaparib combination; **E** ROS level in HMVP2 and 22Rv1 cells after treatment with combination of Cyst(e)inase and Olaparib. ***P < 0.001, ****P < 0.0001, ns = not significant. One-Way ANOVA followed by Dunnett's multiple comparison test. *n* = 3 independent experiments

The effect of combining Cysteine depletion with PARP1 inhibition is shown in Fig. 4. For these experiments, Cyst(e)inase treatment was combined Olaparib with the protocol shown in Fig. 4A. As shown in Fig. 4B, treatment with the combination of Cyst(e)inase and Olaparib for 24 h significantly increased the levels of γH2AX/53BP1 co-staining. Quantitation showed the percent of cells positive for γH2AX/53BP1 co-localization (Fig. 4C) was significantly higher in the combination treatment group (79% for HMVP2 and 76% for 22Rv1) compared to control cells or cells treated with either agent alone. Western blot analysis (Supplemental Fig. 1) showed a significant increase in γH2AX in both cell lines after treatment with the combination of Cyst(e)inase and Olaparib. The combination of Cyst(e)inase and Olaparib also showed a significant increase in pChk1 in both cell lines compared to the individual agents when used alone (see again Supplemental Fig. 1). As shown in Fig. 4D, the combination of Cyst(e)inase and Olaparib synergistically (evaluated again using the Bliss index plot) decreased survival of both HMVP2 and 22Rv1 cell lines. We further investigated the effect of the combination of Cyst(e)inase plus Olaparib on ROS production and found that treatment of Cyst(e)inase alone but not Olaparib alone produced a significant increase in ROS levels (Fig. 4E). These data indicate that Olaparib treatment induced DNA DSBs independent of ROS levels which is consistent with its mechanism of PARP inhibition.

### Cyst(e)inase in combination with Auranofin or Olaparib synergistically reduces growth of PCa cells in xenograft tumor models

**Fig. 5:**
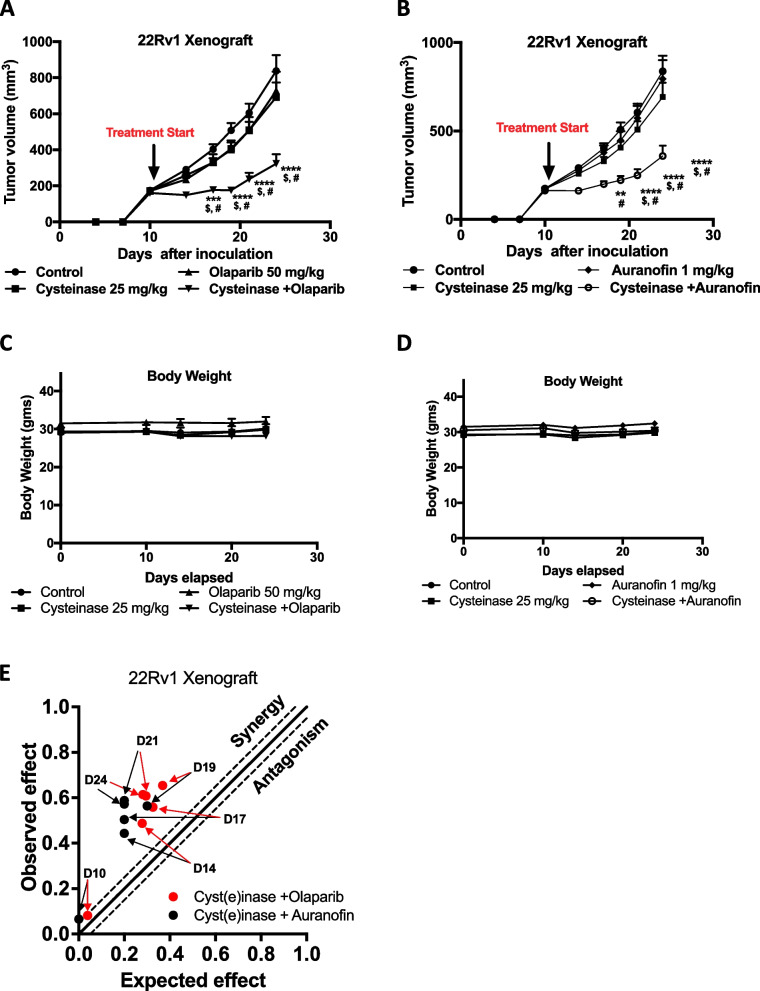
Cyst(e)inase in combination with Olaparib or Auranofin synergistically inhibits growth of PCa xenograft without toxicity. Growth of xenografted 22Rv1 prostate tumors in male nude mice. **A** Data represents Mean ± SEM of both flank tumors in group of mice treated with vehicle control (*n* = 14), Cyst(e)inase (*n* = 15), Olaparib (*n* = 14), or Cyst(e)inase + Olaparib (*n* = 14). Two-Way repeated measure ANOVA followed by Bonferroni’s multiple comparison test. ****P < 0.0001, compared to control; # and $, *P* < 0.01, compared to Cyst(e)inase or Olaparib alone, respectively; **B** Data represents Mean ± SEM of both flank tumors of group of mice treated with vehicle control (*n* = 14), Cyst(e)inase 25 mg/kg (*n* = 15), Auranofin 1 mg/kg (*n* = 14), Cyst(e)inase 25 mg/kg + Auranofin 1 mg/kg (*n* = 14) Two-Way repeated measure ANOVA followed by Bonferroni’s multiple comparison test. **P < 0.01, ****P < 0.0001 compared to control; # and $, *P* < 0.05, compared to Cyst(e)inase or Olaparib alone, respectively; **C, D** Average body weight per mouse from xenograft tumor studies, (A) and (B), respectively; **E** Synergistic reduction of tumor growth with combination of Cyst(e)inase + Olaparib and Cyst(e)inase + Auranofin. The data points above the line indicates Bliss index value from 22Rv1 xenograft tumor from (**A**) and (**B**), showing synergistic effects

Given that the combination of Cyst(e)inase together with Auranofin or Olaparib showed synergistic effects in PCa cells in vitro, we examined whether this effect could be recapitulated in vivo on tumor growth. As shown in Fig. 5A, administration of Cyst(e)inase (25 mg/kg, 2x/week) or Olaparib (50 mg/kg, 5x/week) alone did not show significant reduction of 22Rv1 tumor growth in vivo. However, the combination of Cyst(e)inase + Olaparib produced a strong synergistic effect with significant reduction of tumor growth without any noticeable adverse side effects. In addition, the combination of Cyst(e)inase and Auranofin was evaluated using the same 22Rv1 xenograft tumor model. As shown in Fig. 5B, combining Auranofin with Cyst(e)inase also significantly inhibited tumor growth. No differences were observed in body weight or food consumption in the various groups of mice throughout the studies (Fig. 5C and D and Supplemental Fig. 2A and B) in both combinations. Synergistic inhibitory effects of these two combinations on tumor growth is shown by the Bliss index plot (Fig. 5E) which indicates that both the Cyst(e)inase + Olaparib and the Cyst(e)inase + Auranofin combination produced strong synergistic effects for reduction of tumor growth.

### BRCA2 deficiency sensitizes PCa cells to Cyst(e)inase treatment and enhances DNA DSB formation

**Fig. 6:**
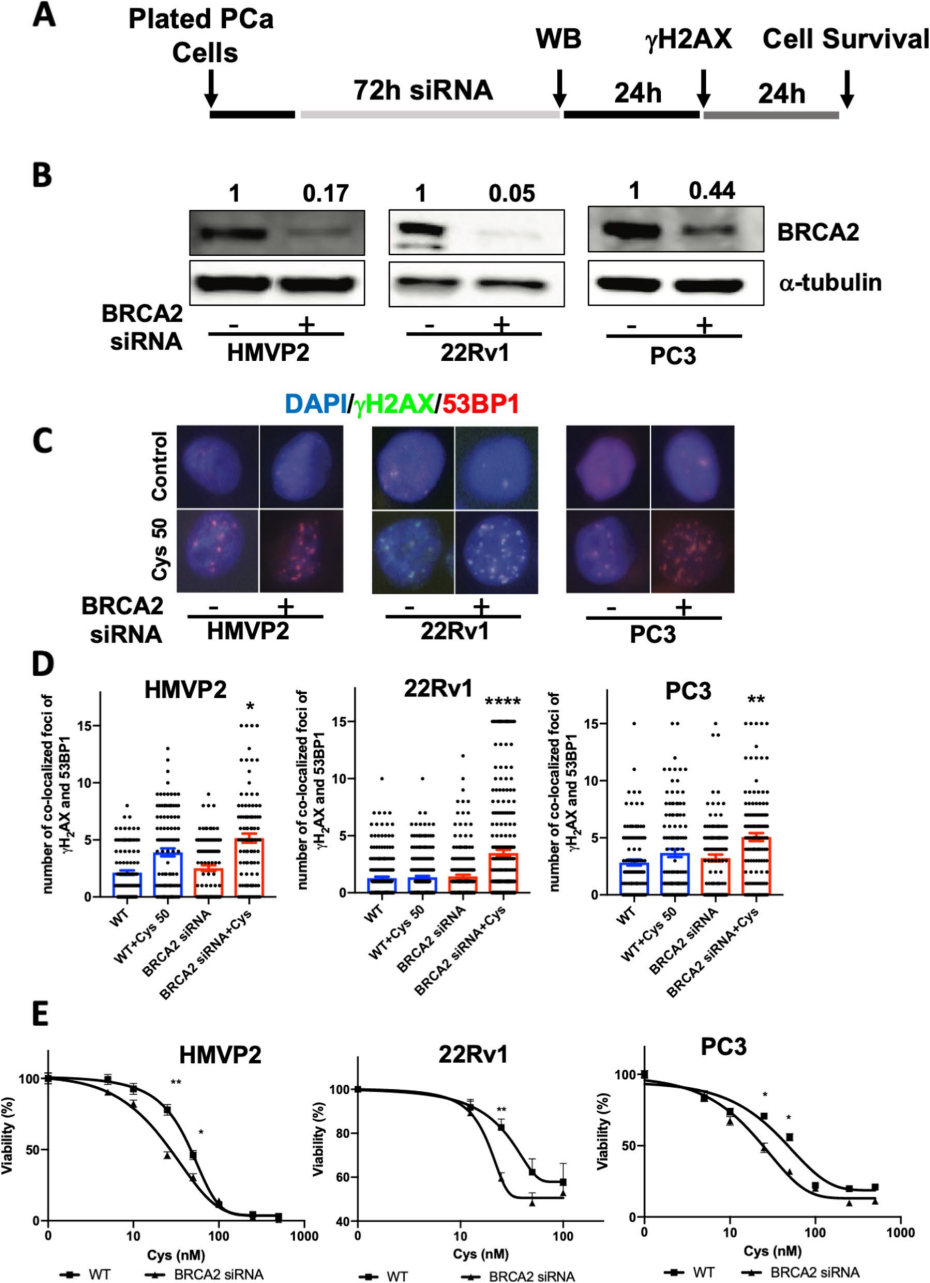
BRCA2 deficiency increases sensitivity of Cyst(e)inase treatment. **A** Schematics of BRCA2 knockdown and Cyst(e)inase treatment procedure; **B** HMVP2, 22Rv1 and PC3 cells were transfected with BRCA2 siRNA (ON-TARGET plus siRNA: SMART pool; Cat# L-003462–00-0005, and L-042993–00-0005) for 72 h and BRCA2 knockdown was determined by Western blot (α-tubulin as loading control); **C** Representative image of γH2AX/53BP1 co-localization in BRCA2 proficient and deficient (siRNA BRCA2) cells after treatment with vehicle or Cyst(e)inase (50 nM). **D** Percent of BRCA2 proficient and deficient cells positive for co-localization of γH2AX with 53BP1; **E** BRCA2 proficient and deficient cells were treated with different concentration of Cyst(e)inase for 72 h and cell viability was measured by crystal violet assay. More than 110 cells were scored from each group for γH2AX/53BP1 co-localization. *P < 0.05, **P < 0.01 and ****P < 0.0001; Student’s t test (D) or One way ANOVA followed by Dunnett's multiple comparison test (E). *n* = 3 independent experiments

BRCA2 protein plays an important role in repair of DNA DSBs via homologous recombination (HR) [19, 20]. In light of the data showing that Cyst(e)inase treatment produced significant DNA damage in the form of DSBs, we next examined whether depleting BRCA2 in PCa cells could sensitize them to Cyst(e)inase treatment. Figure 6A shows the protocol for these experiments using siRNA knockdown (KD) of BRCA2. As shown in Fig. 6B, siRNA mediated KD of BRCA2 was confirmed in multiple PCa cell lines with approximately 50–95% KD compared to control siRNA. Cyst(e)inase treatment of BRCA2 KD PCa cells showed increased number of γH2AX/53BP1 co-localized foci (Fig. 6C and D). PCa cells with BRCA2 KD also showed increased sensitivity to Cyst(e)inase treatment with reduced survival compared to corresponding BRCA2 proficient PCa cells treated with the enzyme (Fig. 6E). These data with BRACA2 KD in PCa cells confirms a role for HR in repair of Cyst(e)inase mediated DNA DSBs.

### Anti-PD-L1/Cyst(e)inase co-treatment increases tumor infiltrating T-cells and synergistically inhibits PCa growth

**Fig. 7:**
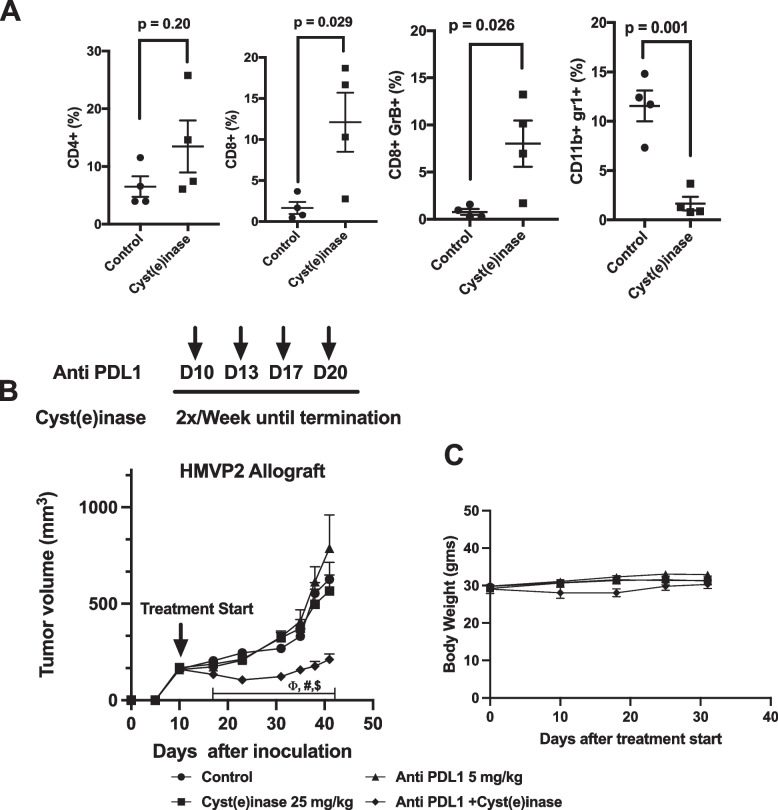
Cyst(e)inase increases cytotoxic T cells, reduces immunosuppressive myeloid cell populations and produces synergistic inhibition of HMVP2 allograft tumor growth. **A,** Allografted HMVP2 prostate tumors in syngeneic FVB mice treated with vehicle control or Cyst(e)inase (100 mg/kg, 2x/week, i.p.) for 2 weeks. CD4 + T-cells; CD8 + T-cells; CD8 + GranzymeB + T-cells; CD11b + gr1 + myeloid cells in the tumor (Students t-test). **B**, Growth of allografted HMVP2 prostate tumors in male syngeneic FVB mice. Data represents Mean ± SEM of both flank tumors in group of mice treated with vehicle control (*n* = 14), Cyst(e)inase (*n* = 16), Anti PDL1 (*n* = 14), or Cyst(e)inase + Anti PDL1 (*n* = 13). Two-Way repeated measure ANOVA followed by Bonferroni’s multiple comparison test. *P* < 0.05, ϕ compared to control; # compared to Cyst(e)inase and $, compared to Anti PDL1. **C**, Average body weight per mouse from allograft tumor study (B)

A recent study with Cyst(e)inase showed that it produced a strong synergistic effect when used in combination with anti-PD-L1 in treating a murine model of ovarian cancer (ID8, BRCA2^wt^) [21]. Anti-PD-L1/Cyst(e)inase co-treatment significantly increased the population of tumor infiltrating T-cells and elicited ferroptotic cell death in this tumor model [21]. Recent studies have suggested that ferroptosis induction may hold promise for the treatment of PCa [22]. To investigate this hypothesis, we evaluated lymphocyte panels from HMVP2 allograft tumors treated with Cyst(e)inase (100 mg/kg, 2x/ week) for two weeks. As shown in Fig. 7A, we observed that Cyst(e)inase treatment led to a modest but non-significant increase in CD4 + T-cells. In contrast, Cyst(e)inase treatment significantly increased CD8 + T-cells and CD8 + GrB + cytotoxic T cells. Cyst(e)inase treatment also significantly reduced the population of CD11b + gr1 + myeloid derived suppressor cells, an effect whose significance for anti-cancer activity was recently reported to be tumor-context dependent [23]. Furthermore, treatment of HMVP2 allograft tumors with 25 mg/kg Cyst(e)inase led to a significantly increased level of tumor associated PD-L1 protein compared to tumors from mice in the control group (Supplemental Fig. 3). Based on these data, we performed a tumor experiment combining Cyst(e)inase with anti-PD-L1 antibody in the HMVP2 mouse allograft tumor model. As shown in Fig. 7B, HMVP2 allograft tumors were generated and treatment started at day 10. Notably, Cyst(e)inase (25 mg/kg, twice weekly) or anti-PD-L1 (5 mg/kg given twice-weekly for two weeks) alone had no effect on HMVP2 tumor growth. In contrast, the combination of Cyst(e)inase + anti-PD-L1 synergistically reduced tumor growth in this immune competent PCa tumor model without significant effects on body weight (Fig. 7C).

## Discussion

Although there has been significant progress in treatment options for PCa in recent years, PCa still represents one of the major cancers leading to morbidity and mortality in men. Lack of good treatment options in advanced PCa as well as the emergence of resistance to existing treatment regimens necessitates the identification and development of novel approaches for improving therapy outcomes. Because many cancer cells, including PCa, have high basal levels of ROS, the use of ROS-generating agents to selectively kill cancer cells has gained attention as an alternative approach for cancer treatment [9, 24–27]. In previous studies, we demonstrated that Cyst(e)inase treatment led to depletion of intracellular GSH and death of PCa cells in vitro and in vivo by induction of higher ROS levels [13]. The current data show that Cyst(e)inase-induced ROS in PCa cells causes oxidative DNA damage leading to DNA DSBs. In addition, strategies that further increased production of ROS by blocking alternate antioxidant pathways (i.e., TXNR) or compromising DNA repair (PARP inhibition; BRCA2 mutation) exacerbated this DNA damage leading to synergistic PCa cell killing and inhibition of PCa tumor growth. In addition to these results, systemic depletion of L-Cys/CSSC with Cyst(e)inase led to changes in tumor infiltrating immune cells that sensitized PCa cells to treatment with anti-PD-L1. Collectively, the current results demonstrate that combination of several types of agents with Cyst(e)inase represents potential novel approaches for treating PCa.

Several studies have shown that simultaneously targeting multiple antioxidant systems (for instance both GSH and TXNR systems) can potentially provide better therapeutic outcomes and even synergistic combinatorial effects for cancer treatment in preclinical models [28–30]. In our earlier studies, we showed that Cyst(e)inase in combination with BSO (GSH synthesis inhibitor) produced synergistic induction of ROS and tumor cell killing [13]. In addition, Cyst(e)inase + curcumin (the latter an irreversible inhibitor of TXNR) also produced synergistic induction of ROS and inhibition of tumor growth in vivo [13]. In the current study, we found that Cyst(e)inase treatment increased clustered oxidative DNA damage and DNA DSBs in PCa cells (Figs. 1 and 2). Combining Cyst(e)inase with a specific TXNR inhibitor, Auranofin, further increased DNA DSBs in PCa cells (Fig. 3). Since the combination of Cyst(e)inase and Auranofin blocks both GSH and TXNR antioxidant systems, this leads to overproduction of ROS and increased levels of DNA DSBs seen in PCa cells treated with this combination. These data further confirm that targeting both GSH and TXNR antioxidant systems simultaneously can achieve synergistic cancer cell killing through increased oxidative DNA damage and formation of DNA DSBs in PCa cells.

PARP is essential for various cellular processes including DNA replication, recombination and repair and PARP inhibitors show efficacy in treating various solid tumors [31, 32]. Several PARP inhibitors have shown promising clinical outcomes in treating metastatic CRPC (mCRPC) [32, 33]. Olaparib is the most widely studied PARP inhibitor for various cancers and inhibits the enzymatic activity of PARP and DNA repair processes [34]. In the current study, Cyst(e)inase in combination with Olaparib treatment showed synergistic killing of PCa cells in vitro and tumor growth in vivo. The combination of Cyst(e)inase + Olaparib also significantly increased DNA DSBs without further induction of ROS (Fig. 4) indicating a mechanism of action different than the combination of Cyst(e)inase + Auranofin. These results demonstrate that Olaparib in the presence of Cyst(e)inase decreased repair of DNA damage via inhibition of PARP, leading to further accumulation of SSBs and DSBs and synergistic PCa cell killing.

Analysis of The Cancer Genome Atlas (TCGA) reveals that 19% of primary prostate cancers have mutations in DNA repair genes [35]. Robinson et al. reported that metastatic PCa tissue samples had 23% defects in DNA repair genes including BRCA2 [36]. Mutations in BRCA2 are observed in 13.3% of primary prostate tumors [36]. It is already established that the mutation of BRCA2 is associated with unrepaired DSBs leading to genomic instability and cancer progression [37, 38]. Notably, prostate tumors with HR gene mutations are sensitive to PARP inhibitors [32, 33]. The current data demonstrate that treatment with Cyst(e)inase significantly increased DNA DSBs and decreased cell survival in BRCA2 deficient PCa cell lines compared to the parental cell lines with intact BRCA2, indicating that compromised HR repair leads to greater accumulation of DSBs and subsequent reduction of PCa cell survival. These results are consistent with previously published studies which reported that PARP inhibition and BRCA1/2 deficiency produced synthetic lethality [39, 40]. Recent clinical trials showed promising results with PARP1 inhibitors for treating advanced PCa in combination with DNA damaging chemotherapies [32, 41, 42]. Based on these results, two PARP inhibitors received FDA clinical approval for men with mCRPC. In this regard, oral rucaparib (Rubraca) was approved for the treatment of mCRPC patients with a BRCA mutation and who had been previously treated with androgen receptor-directed therapy and a taxane-based chemotherapy (https://www.fda.gov/drugs/fda-grants-accelerated-approval-rucaparib-brca-mutated-metastatic-castration-resistant-prostate). Olaparib (Lynparza) was also approved for the treatment of patients with mCRPC and HR repair gene mutations who have progressed following prior treatment with enzalutamide (Xtandi) or abiraterone (Zytiga) (https://www.fda.gov/drugs/drug-approvals-and-databases/fda-approves-olaparib-hrr-gene-mutated-metastatic-castration-resistant-prostate-cancer). Although PARP inhibitors show efficacy and are well tolerated in many BRCA1/2 mutated cancers, a fraction of tumors become resistant and some patients do not respond well. Several mechanisms are proposed for this resistance including restoration of the defects in HR such as reversal of BRCA1/2 truncation mutation, rewiring of DNA damage repair activity and hyperactivity of BRCA1/2 variants [43]. In addition, increased drug efflux by p-glycoprotein are also associated with the development of resistance to PARP inhibitors [43]. Our current results with Cyst(e)inase suggest a potential novel alternative treatment strategy to overcoming PARP inhibitor resistance in PCa patients carrying BRCA mutation.

PD-L1 is selectively expressed on many tumors [44, 45] and on cells within the tumor microenvironment in response to inflammatory stimuli [46]. PD-L1 inhibits cytokine production and the cytolytic activity of PD-1 + , tumor-infiltrating CD4 + and CD8 + T cells [44, 47]; conversely, inhibition of the PD‐1/PD‐L1 axis has produced impressive response rates in various cancer types. While single agent immune checkpoint therapy for PCa has not been effective, anti-PD1/Olaparib combination therapy is demonstrating promising responses in phase I/II clinical trials [42]. Likewise, preclinical studies have shown that DNA damaging chemotherapy treatment enhances PCa response to immunotherapy [48] and in particular, Olaparib administration upregulates PD-L1 expression in other tumor models [49, 50].

Recent findings demonstrate that Cyst(e)inase treatment in combination with anti-PD-L1 checkpoint blockade synergistically enhanced T cell-mediated anti-tumor immunity, elevating tumoral lipid ROS and increasing populations of IFNγ/TNF expressing CD8 + /CD4 + T cells in an ovarian cancer model [21]. Notably, we found that Cyst(e)inase treatment of HMVP2 mouse PCa tumor allografts significantly increased the number of CD8 + T-cells and reduced the number of myeloid derived suppressor cells (see Fig. 7A). Cyst(e)inase treatment also increased the protein level of PD-L1 in HMVP2 allograft tumors. The combinaton of Cyst(e)inase and anti-PD-L1 treatment significantly and synergistically inhibited HMVP2 PCa tumor growth in vivo. Thus, depletion of L-Cys/CSSC also increased sensitivity to anti-PDL1 treatment in this mouse model of PCa and warrants further preclinical and possible clinical investigation.

## Conclusion

The current results demonstrate that depletion of L-Cys/CSSC with a human enzyme, Cyst(e)inase, causes clustered oxidative DNA damage leading to DNA DSBs in PCa cells. The exact mechanism for the formation of DSBs in Cyst(e)inase treated cells remains to be fully elucidated, but they are likely formed during the processing of BER intermediates and are independent of the replication fork as previously discussed [18]. Combinations of Cyst(e)inase with a TXNR inhibitor (Auranofin) or a PARP inhibitor (Olaparib) further increased DNA damage and led to synergistic reduction of cell survival in culture and synergistic reduction in PCa tumor growth in xenograft tumor models. BRCA2 deficiency also increased the sensitivity of PCa cells to Cyst(e)inase-induced DNA DSBs and cell survival inhibition. The current results also demonstrated that L-Cys/CSSC depletion sensitized PCa cells to immune checkpoint inhibition (ICI) using anti-PD-L1 antibody in part through modulation of immune cells in the tumor microenvironment. Increased DNA damage caused by Cyst(e)inase treatment may also play a role in sensitizing PCa cells to ICI treatment. Collectively, the current data further demonstrate the potential of L-Cys/CSSC depletion using an engineered human therapeutic enzyme, particularly in combination with other agents, as a novel therapeutic strategy for PCa. Future studies will examine the efficacy of Cyst(e)inase combinations in primary mouse tumor models and humanized PDX models to further develop potential clinical applications.

## Supplementary Information

**Additional file 1: Supplemental Figure 1.** Alteration of DNA damage related proteins after treatment of combination of Cyst(e)inase with Olaparib or Auranofin. Cells were treated with indicated concentrations of Cys, Ola, Aur or their combinations for 24h and subjected to Westren blot analyses of total and phospho proteins. **Supplemental Figure 2.** Cyst(e)inase synergistically inhibits growth of 22Rv1 PCa xenografts in combination with Olaparib or Auranofin without toxicity. A, Average food consumptions in 22Rv1 xenograft tumors study in male nude mice treated with A, vehicle control, Cys, Ola, Cys+Ola or B, vehicle control, Cys, Aur, Cys+Aur. **Supplemental Figure 3.** Cyst(e)inase treatment of HMVP2 allograft tumors leads to increased expression of PD-L1. Protein was isolated from pooled tumors of control (vehicle treated) and Cyst(e)inase treated mice and subjected to Western blot analyses with anti-PD-L1 antibody (Abcam, 1:1000 dilution) and further processed as described in the Methods section.

## Data Availability

The data generated in this study are available upon request from the corresponding author (JD).

## Abbreviations

PCa: Prostate cancer
FACS: Fluorescence-Activated Cell Sorting
CRPC: Castrate resistant prostate cancer
SSBs: Single strand breaks
DSBs: Double strand breaks
ROS: Reactive oxygen species
GSH: Glutathione
L-Cys: L-cysteine
CSSC: Cystine
TXNR: Thioredoxin reductase
PARP: Poly (ADP-ribose) polymerase
PD-L1: Programmed cell death ligand -1
PFGE: Pulse field gel electrophoresis
HR: Homologous recombination
KD: Knockdown
TCGA: The Cancer Genome Atlas
ICI: Immune checkpoint inhibition
BER: Base excision repair
