# Supplementary material for: Cysteine depletion sensitizes prostate cancer cells to agents that enhance DNA damage and to immune checkpoint inhibition
Source: J Exp Clin Cancer Res. 2023 May 11;42:119. doi: 10.1186/s13046-023-02677-2 (PMC10173527; doi:10.1186/s13046-023-02677-2)
Supplement: Supplementary file 1 — Additional file 1: Supplemental Figure 1. Alteration of DNA damage related proteins after treatment of combination of Cyst(e)inase with Olaparib or Auranofin. Cells were treated with indicated concentrations of Cys, Ola, Aur or their combinations for 24h and subjected to Westren blot analyses of total and phospho proteins. Supplemental Figure 2. Cyst(e)inase synergistically inhibits growth of 22Rv1 PCa xenografts in combination with Olaparib or Auranofin without toxicity. A, Average food consumptions in 22Rv1 xenograft tumors study in male nude mice treated with A, vehicle control, Cys, Ola, Cys+Ola or B, vehicle control, Cys, Aur, Cys+Aur. Supplemental Figure 3. Cyst(e)inase treatment of HMVP2 allograft tumors leads to increased expression of PD-L1. Protein was isolated from pooled tumors of control (vehicle treated) and Cyst(e)inase treated mice and subjected to Western blot analyses with anti-PD-L1 antibody (Abcam, 1:1000 dilution) and further processed as described in the Methods section. [file 13046_2023_2677_MOESM1_ESM.pdf]

Figure S1

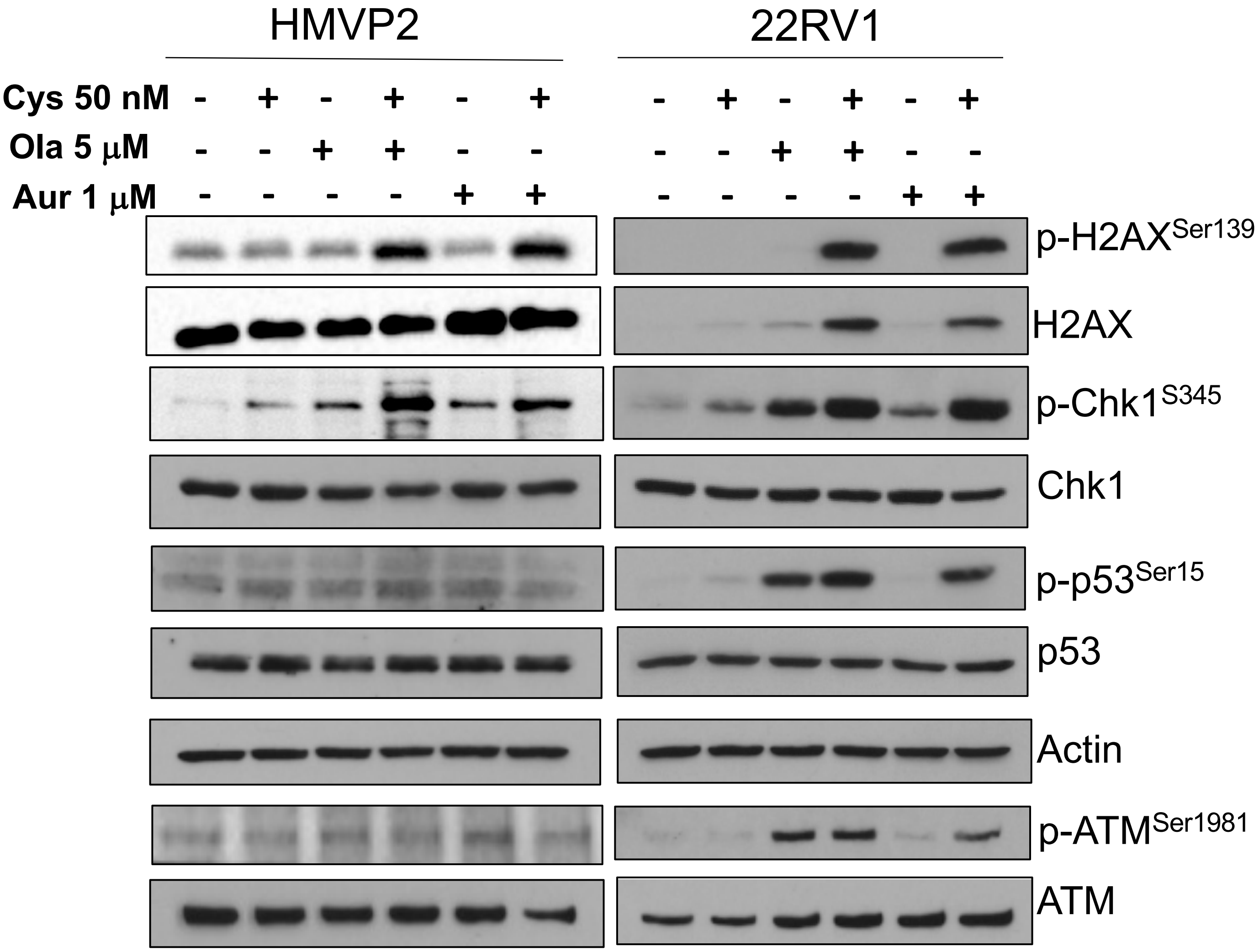

Supplemental Figure 1. Alteration of DNA damage related proteins after treatment of combination of Cyst(e)inase with Olaparib or Auranofin. Cells were treated with indicated concentrations of Cys, Ola, Aur or their combinations for 24h and subjected to Westren blot analyses of total and phospho proteins.

Figure S2

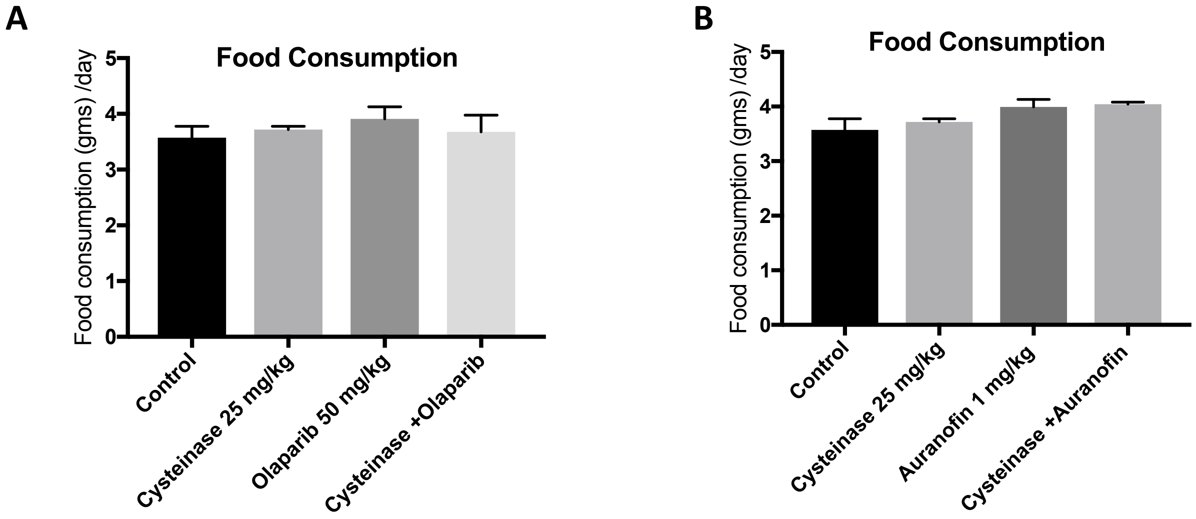

Supplemental Figure 2. Cyst(e)inase synergistically inhibits growth of 22Rv1 PCa xenografts in combination with Olaparib or Auranofin without toxicity. A, Average food consumptions in 22Rv1 xenograft tumors study in male nude mice treated with **A**, vehicle control, Cys, Ola, Cys+Ola or **B**, vehicle control, Cys, Aur, Cys+Aur.

Figure S3

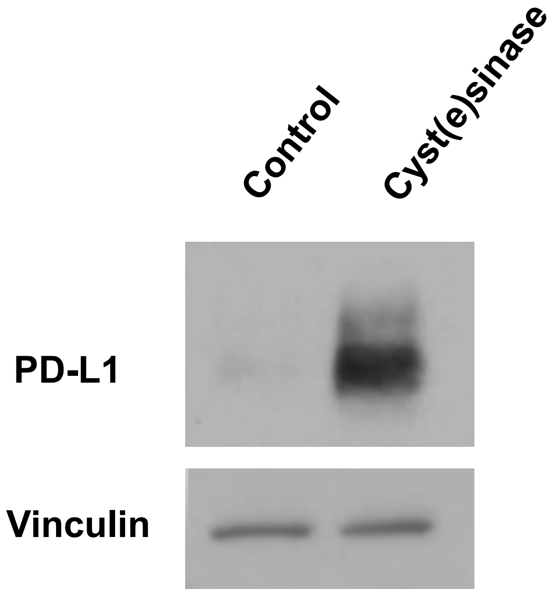

Supplemental Figure 3: **Cyst(e)inase treatment of HMVP2 allograft tumors leads to increased expression of PD-L1**. Protein was isolated from pooled tumors of control (vehicle treated) and Cyst(e)inase treated mice and subjected to Western blot analyses with anti-PD-L1 antibody (Abcam, 1:1000 dilution) and further processed as described in the Methods section.
